# Longitudinal associations of *in utero *and early life near-roadway air pollution with trajectories of childhood body mass index

**DOI:** 10.1186/s12940-018-0409-7

**Published:** 2018-09-14

**Authors:** Jeniffer S. Kim, Tanya L. Alderete, Zhanghua Chen, Fred Lurmann, Ed Rappaport, Rima Habre, Kiros Berhane, Frank D. Gilliland

**Affiliations:** 1 Department of Preventive Medicine, Division of Environmental Health, Keck School of Medicine, Southern California Environmental Health Sciences Center, University of Southern California, 2001 N. Soto Street, Los Angeles, CA 90032 USA; 2 Sonoma Technology Inc., Petaluma, CA USA; 3 Department of Integrative Physiology, University of Colorado at Boulder, Boulder, CO USA

**Keywords:** Near-roadway air pollution, *In utero* exposures, Early life exposures, Childhood body mass index, Childhood obesity

## Abstract

**Background:**

Evidence suggests that childhood near-roadway air pollution (NRAP) exposures contribute to increased body mass index (BMI); however, effects of NRAP exposure during the vulnerable periods including *in utero* and first year of life have yet to be established. In this study, we examined whether exposure to elevated concentrations of NRAP during *in utero* and/or first year of life increase childhood BMI growth.

**Methods:**

Participants in the Children’s Health Study enrolled from 2002 to 2003 with annual visits over a four-year period and who changed residences before study entry were included (*n* = 2318). Annual height and weight were measured and lifetime residential NRAP exposures including *in utero* and first year of life periods were estimated by nitrogen oxides (NO_x_) using the California line-source dispersion model. Linear mixed effects models assessed *in utero* or first year near-road freeway and non-freeway NO_x_ exposures and BMI growth after adjusting for age, sex, race/ethnicity, parental education, Spanish questionnaire, and later childhood near-road NO_x_ exposure.

**Results:**

A two-standard deviation difference in first year of life near-road freeway NO_x_ exposure was associated with a 0.1 kg/m^2^ (95% confidence interval (CI): 0.03, 0.2) faster increase in BMI growth per year and a 0.5 kg/m^2^ (95% CI: 0.02, 0.9) higher attained BMI at age 10 years.

**Conclusions:**

Higher exposure to early life NRAP increased the rate of change of childhood BMI and resulted in a higher attained BMI at age 10 years that were independent of later childhood exposures. These findings suggest that elevated early life NRAP exposures contribute to increased obesity risk in children.

**Electronic supplementary material:**

The online version of this article (10.1186/s12940-018-0409-7) contains supplementary material, which is available to authorized users.

## Background

In the United States, approximately 32% of children 2 to 19 years were overweight or obese in 2011–2012 [1]. High prevalence of childhood obesity present significant clinical and public health problems since obese children are more likely to become obese adults [2, 3] and are at a greater risk for developing type 2 diabetes and cardiovascular disease [4–7]. Despite decades of diet and physical activity interventions, the prevalence of childhood obesity remains high [8]. Previous studies have shown that increased near-roadway air pollution (NRAP) exposure [9, 10] and increased traffic density [9, 11] during childhood contributes to increased obesity risk in children. These findings suggest that modifiable environmental factors such as air pollution exposures may be contributing to the obesity epidemic [9, 12].

Longitudinal studies have shown that increased mid-childhood NRAP exposure and traffic density are associated with substantially increased body mass index (BMI) growth and a higher level of attained BMI at ages 18 [11] and 10 years [9]. Furthermore, increased childhood NRAP exposures, secondhand smoke (SHS), and maternal smoking during pregnancy were associated with increased BMI growth and a higher BMI at age 18 years [10]. To date, few studies have examined the effects of *in utero* and early childhood NRAP exposure on childhood BMI growth. Beyond mid-childhood exposures, early life periods like *in utero* and first year of life represent critical windows of air pollution exposure that may significantly alter childhood growth trajectories. One epidemiological study found that increased *in utero* ambient polycyclic aromatic hydrocarbon exposure, a marker for NRAP, was associated with higher BMI z-scores at age 5 and 7 years [13]. Additionally, recent data from the Boston Birth Cohort showed that * in utero* and early life exposures to ambient, fine particulate matter ≤2.5 μm in diameter (PM_2.5_) were significantly associated with increased risk of childhood overweight or obesity in children 2–9 years of age [14]. Although these studies suggest that elevated NRAP exposures during early life and childhood may increase future obesity risk, studies have not been entirely consistent as a European birth cohort recently reported no association of first 4 years of life NRAP exposure and childhood obesity at 4 years and 8 years of age [15].

Despite mixed findings*,* *in utero *and first year of life are periods of rapid growth that are highly susceptible to environmental influences [16]. Lasting effects of * in utero* and early life environmental exposures on childhood growth trajectories have been shown, and these early life stages are critical periods for the development of obesity [16, 17]. For example, past studies have reported associations of increased *in utero* air pollution exposure and restricted fetal growth resulting in low birth weight (LBW) in full term babies [18–20]. Consequently, term babies with LBW have been shown to have postnatal catch-up growth which in turn has been associated with higher weight gain through infancy and childhood [21, 22]. Because *in utero* and first year of life are important developmental periods that influence growth, increased NRAP exposure during these critical periods may be contributing to future obesity risk through altered growth trajectories resulting in faster childhood BMI growth. The objective of this study was to examine the relationships between *in utero* and first year of life NRAP exposures with longitudinal measurements of BMI in a subset of Southern California children enrolled in the Children’s Health Study (CHS). We hypothesized that higher * in utero* and/or first year of life NRAP exposure is associated with a faster increase in BMI growth over a 4-year follow up period and a higher attained BMI at age 10 years, and that these early life NRAP effects are independent of NRAP exposures later in childhood.

## Methods

### Study design

**Fig. 1:**
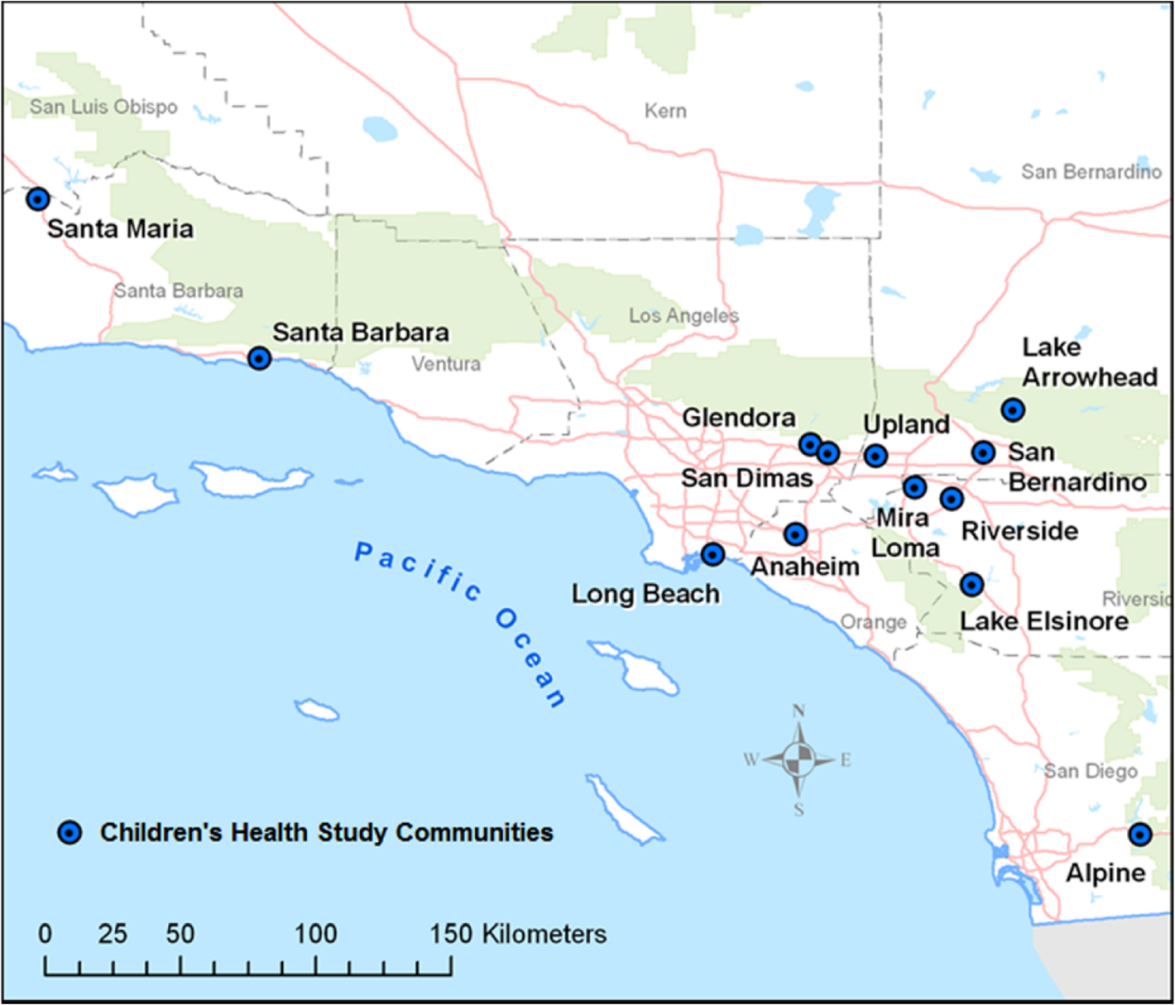
Map of Children’s Health Study Communities

From 2002 to 2003, a cohort of kindergarten and first grade children were recruited from 45 public schools across 13 Southern California communities (Fig. 1). Details of CHS recruitment methods have been previously presented [23]. Informed consents were obtained from parents and assents from children. This study was reviewed and approved by the Institutional Review Board at the University of Southern California.

Height and weight were measured at baseline and at each subsequent, annual school visit. A trained technician measured height to the nearest centimeter and weight to the nearest pound (0.45 kg) of each child without shoes and with daily calibrations of the weight scale. BMI was calculated by weight/height^2^ (kilograms/meters^2^) and overweight and obese were defined using the Centers for Disease Control and Prevention (CDC) age-and sex-specific growth charts [24]. Parents completed baseline and yearly follow-up questionnaires pertaining to sociodemographic factors, characteristics of the home, and other covariates that were explored as potential confounders. These included age, sex, race/ethnicity, self-reported premature birth, maternal smoking during pregnancy, residential SHS, lifetime history of asthma, parental education (marker for socioeconomic status), if baseline questionnaire was completed in Spanish (marker for recent immigration status), and child’s participation in team sports during the past year. A subset of children had birth weight and gestational age at birth which were extracted from the California vital statistics records. These data were obtained using the CDC LINKPLUS program which matched CHS children to the state’s vital records database using several variables including child’s name, sex, birthdate, as well as, mother’s name, birthdate, zip code, and father’s name.

### NRAP exposures

Lifetime residential history was collected from all CHS participants at study enrollment and at each annual CHS visit via questionnaires, including move in and move out dates for each residence. NRAP exposures were estimated based on street level geocoded, residential locations from *in utero* to the most current follow-up date, which gave estimates of NRAP exposure for* in utero*, first year of life, and childhood periods. For *in utero* exposures, street address level data from study questionnaires (74.8%) and birth certificates (25.2%) were used. Residential addresses were uploaded to the ESRI geocoding database and software (ESRI Inc., Redlands, CA http://www.esri.com), geocoded to street level using the software, and assigned latitude and longitude coordinates. Google Earth and the Texas A&M geocoder [25] were also used to assign coordinates for a small number of problematic residential addresses.

The California line-source dispersion model (CALINE4) was used to estimate concentrations of traffic-related nitrogen oxides (NO_x_) for freeway and non-freeway roads using EMFAC2011 (for 1994–1999) and EMFAC2014 (for > 1999) vehicle emission rates, traffic volume, road geometry and meteorological conditions, including wind speed and direction, pollution mixing heights, and atmospheric stability [26]. Our roadway information is classified according to Feature Class Codes (FCC) which includes (1) primary highways with limited access (freeways), (2) primary roads without limited access, (3) secondary and connecting roads, and (4) local, neighborhood, and rural roads. Annual average traffic volumes from imbedded loop sensors provide 100% coverage for freeways and 95% coverage for state highways (Class 2). The freeways have data for each link between interchanges whereas data for Class 2 roads is often extrapolated for longer distances to provide full coverage for all links. About 5% of smaller roads have traffic volume measurements and because these measurements are often on higher volume roads within their class, we assign the 25th percentile of annual traffic volume to all roads in the corresponding class. Exposure to NRAP was modeled using monthly average CALINE4 estimated NO_x_ concentrations from freeway and non-freeway sources within 5 km of the residential coordinates during the *in utero*, first year of life, and mid-childhood periods. *In utero* near-road NO_x_ exposure was defined as the nine-month average exposure prior to birth and first year of life near-road NO_x_ exposure was defined as the twelve-month average exposure after birth. Lastly, to account for childhood near-road NO_x_ exposures beyond the early life periods, childhood NRAP exposure was calculated as the average near-road NO_x_ exposure from 13 months of age through the 4-year study follow up period. Traffic pollutants are a complex mixture of gases and particles that include NO_x_, carbon monoxide, elemental carbon, particulate matter, organic compounds, and polycyclic aromatic hydrocarbons amongst others. These NRAP exposures reflect increases in local vehicle emissions beyond background ambient levels. Therefore, NRAP was modeled using near-road NO_x_ from freeway and non-freeway roadways as a marker for traffic pollution as this measure is highly correlated with other pollutants estimated by CALINE4.

### Statistical methods

Linear mixed effects models [27, 28] were fitted to estimate longitudinal relationships between BMI trajectory and *in utero* and first year of life NRAP exposures. We examined the associations of *in utero* or first year of life near-road NO_x_ exposure from freeway and non-freeway roads with 1) the rate of change in BMI during 4 years of study follow up and 2) the attained BMI level at age 10 years. Due to the high correlation of *in utero* and first year of life near-road NO_x_ exposures (correlation *r* = 0.8 for freeway, *r* = 0.93 for non-freeway), these two early life periods were analyzed in separate models.

Specifically, the following multi-level mixed effects model was used in the analysis. Let repeated measures of BMI (Y_cij_) with c, i, and j representing the study community, individual, and year of BMI measurement, respectively. Then,

Level 1: Y_cij_ = a_ci_a_ci_ + b_ci_(t_cij_ − C) + γ_1_(E_Fij_ − E_Fi_) + γ_2_W_ij_ + ε_cij._

Level 2a (level): a_ci_ = a_c_ + α_1_E_Ui_ + α_2_E_Fi_ + α_3_Z_i_ + δ_ci_

Level 2b (growth): b_ci_ = β_0_ + β_1_E_Ui_ + β_2_E_Fi_ + β_3_Z_i_ + σ_ci_

Level 3a: a_*c*_ = *α*_0_ + *ε*_c_

In Level 1, *t*_*cij*_ is the age of participants at each visit centered by age C (10 years), and γ_1_ represents cross-sectional association between year to year fluctuations of near-road NO_x_ with follow up BMI measure at each study visit. *E*_*Fij*_ reflects average near-road NO_x_ exposure for the time between each subsequent follow up visit and *E*_*Fi*_is the average childhood near-road NO_x_ exposure from 13 months of age till last height/weight measure in 2006–2007 school year. Importantly, in this analysis we wanted to elucidate associations of *in utero* or first year of life near-road NO_x_ exposures (E_Ui_) with BMI growth independent of childhood near-road NO_x_ exposures. Therefore, the mixed model also includes average childhood near-road NO_x_ exposures (*E*_*Fi*_) while accounting for yearly deviations of near-road NO_x_ during this follow-up period (E_Fij_ − E_Fi_).

In levels 2a and 2b, α_1_ and β_1_ correspond to estimated effects of *in utero* near-road NO_x_ exposure (or first year of life) on attained BMI level at age 10 years and the growth of BMI during the follow-up period, respectively. Whereas, α_2_ and β_2_ correspond to estimated effects of childhood near-road NO_x_ exposure on attained BMI level at age 10 years and the growth of BMI during the childhood period, respectively. Furthermore, α_3_Z_i_ denotes adjustment factors for time-independent covariates at BMI level at age 10, β_3_Z_i_ are adjustment factors for BMI growth during follow-up, ε_cij_, ε_c_, δ_ci_ and σ_ci_ reflect error terms at each level of model to account for the random variations across communities and internal correlations of repeated measures from each subject over time. In addition, nonlinearity of BMI growth trajectory was tested by looking at the slope of BMI growth over time from baseline age 6.5 years to 10 years. We found no deviations from a linear trend as this is a relatively short period of growth ~ 3.5 years, and children in this age range have not yet reached puberty where growth tends to be nonlinear. Therefore, linear BMI growth was considered in the final analysis.

**Fig. 2:**
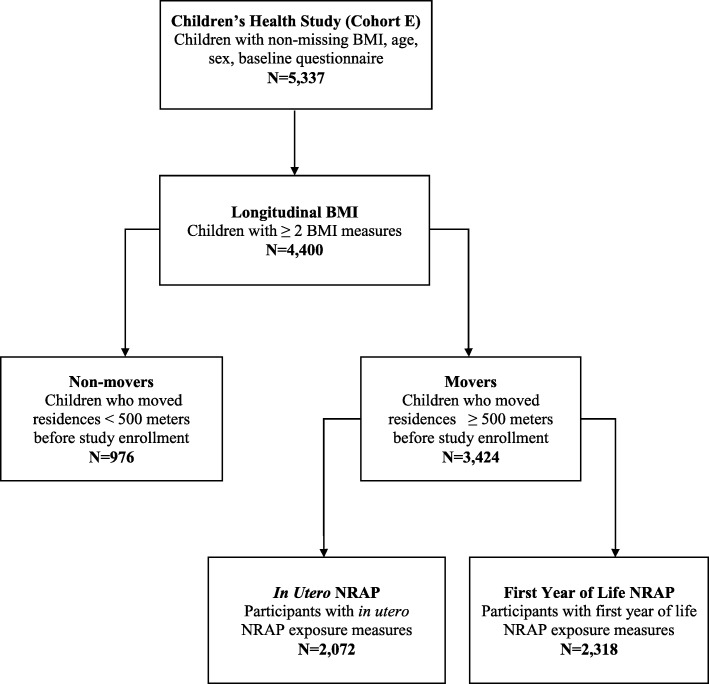
Flow Chart of Children Enrolled in the Children’s Health Study from 2002 to 2003 Included and Excluded from the Current Analysis

The current analysis included 2318 children who: 1) completed a baseline questionnaire, 2) had at least two measures of BMI across the 4-year study follow-up period, 3) had NRAP exposure data for the time windows of interest, and 4) moved homes prior to study enrollment to avoid collinearity between NRAP exposures during the *in utero* or first year of life periods with later childhood exposures (Fig. 2; Additional file 1). Children who lived in the same residence from birth through follow-up had a high correlation of NRAP exposures across each time period, therefore all analyses were restricted to “Movers”. “Movers” were subjects who had a change in address between the *in utero* period and study enrollment that resulted in a move farther than or equal to 500 m. This selection method allowed us to look at later NRAP exposures in childhood in the same model as the early life exposures, *in utero* and first year of life. In the “Movers” group, NRAP exposure periods had lower correlations, which allowed us to use the modeling framework described.

Mixed effects models were used to examine age-adjusted associations between baseline characteristics and BMI growth as well as attained BMI at age 10 years. These characteristics were identified as confounders and included in the final model if they resulted in a ≥ 10% change in the effect estimate for BMI growth or attained BMI at age 10 years. Of the identified potential confounders, age, sex, race/ethnicity, parental education, and Spanish baseline questionnaire were included as confounders in the final model. Furthermore, effect modification by sex and race/ethnicity was tested using interaction terms in the full model. Effect estimates of NRAP exposure on BMI growth as well as attained BMI level at age 10 years are reported for a two-standard deviation (SD) difference in near-road freeway or non-freeway NO_x_ exposures during each exposure window. Statistical significance was based on a two-sided *p* < 0.05. All analyses were performed in SAS, version 9.4 (SAS, Institute, Cary, NC).

## Results

Baseline characteristics are reported in Table 1. At study entry, the mean age was 6.5 years (SD: 0.7) and 50.6% were male. Approximately 29% of children were overweight or obese using CDC growth chart cutoffs [24] with a mean BMI percentile of 60.5 (SD: 30.1) at study entry. Children were predominately Hispanic (56%) or Non-Hispanic White (33%), where 22% of the parents completed the baseline questionnaire in Spanish, which was used a marker of recent immigration status. On average, more than half of the children included in this study had parents with an education level above high school. SHS exposure was relatively low where 7% of mothers smoked during pregnancy and 5% of children lived in homes where someone smoked daily in the presence of the child. At the end of the study follow up period, the mean age of children was 9.5 years (SD: 1.2).

**Table 1:** Baseline characteristics and age-adjusted associations with BMI growth and BMI at age 10 years in children enrolled in the longitudinal Children’s Health Study^a^

Characteristic	n (%)^b^	Associations with BMI growth^c^ (95% CI)	Associations with BMI at Age 10 years^c^ (95% CI)
Overweight/Obesity Status^d^			
Normal	1651 (71.2)	reference	reference
Overweight	320 (13.8)	0.4 (0.3, 0.5)	4.1 (3.8, 4.5)
Obese	347 (15.0)	0.7 (0.7,0.8)	9.0 (8.6, 9.3)
Sex			
Female	1145 (49.4)	reference	reference
Male	1173 (50.6)	0.06 (0.004, 0.1)	0.3 (−0.06, 0.7)
Race/Ethnicity			
White	771 (33.3)	reference	reference
Hispanic	1290 (55.7)	0.3 (0.2, 0.4)	1.9 (1.6, 2.3)
Black	72 (3.1)	0.2 (−0.02, 0.3)	1.2 (0.1, 2.2)
Asian/Pacific Islander	67 (2.9)	0.2 (−0.006, 0.4)	0.9 (−0.2, 2.0)
Other	114 (4.9)	0.2 (0.04, 0.3)	0.7 (−0.2, 1.5)
Parental Education			
Less than high school	410 (18.4)	reference	reference
High school	430 (19.3)	−0.2 (− 0.3, − 0.06)	−1.0 (−1.6, − 0.5)
Above high school	1389 (62.3)	−0.3 (− 0.4, − 0.2)	−2.0 (−2.4, − 1.4)
Spanish Questionnaire^e^			
No	1818 (78.4)	reference	reference
Yes	500 (21.6)	0.2 (0.2, 0.3)	1.7 (1.3, 2.1)
Self-Reported Premature Birth			
No	2003 (88.9)	reference	reference
Yes	251 (11.1)	−0.01 (− 0.1, 0.08)	−0.6 (− 1.1, 0.02)
Maternal Smoking During Pregnancy			
No	2083 (92.7)	reference	reference
Yes	164 (7.3)	−0.01 (− 0.1, 0.1)	− 0.5 (− 1.2, 0.2)
Residential Second-Hand Smoke^f^			
No	2110 (93.2)	reference	reference
Yes, child is home	110 (4.9)	0.1 (0.02, 0.2)	0.3 (0.07, 0.6)
Yes, child is not home	44 (1.9)	0.1 (−0.04, 0.2)	0.2 (− 0.2, 0.5)
Lifetime History of Asthma			
No	1928 (85.1)	reference	reference
Yes	338 (14.9)	−0.04 (− 0.1, 0.008)	0.09 (− 0.08, 0.2)
Organized Team Sport^g^			
No	1141 (57.8)	reference	reference
Yes	832 (42.2)	−0.08 (− 0.1, − 0.04)	−0.2 (− 0.3, − 0.09)

^a^This analysis includes a subset of the Children’s Health Study participants who had available NRAP exposure data for *in utero* or first year of life periods, at least two measures of BMI during study follow up period, completed a baseline questionnaire, and had moved homes at least once before study enrollment

^b^First observation of participant with NRAP exposures (*n* = 2318); variable denominators may differ due to missing values.

^c^Age-adjusted association for each characteristic with BMI growth over study follow up period and attained BMI at age 10 years

^d^Overweight/ Obesity status: Normal is <85th percentile of age-, sex-specific BMI using 2000 CDC growth chart, overweight is 85-95th percentile of age-, sex-specific BMI, obese is ≥95th percentile of age-, sex- specific BMI.

^e^Spanish Questionnaire is if parent filled out baseline questionnaire in Spanish and serves as a surrogate measure for recent immigration.

^f^ Residential second-hand smoke is if anyone living in the child’s home smokes daily inside the home.

^g^Organized team sport is if the child played outdoors in any organized team sport at least twice a week during the past year

Age-adjusted associations between each baseline characteristic and BMI growth trajectory and attained BMI at age 10 years are also shown in Table 1. Briefly, BMI growth through the study period was associated with baseline overweight/obesity status, sex, race/ethnicity, parental education, Spanish questionnaire, residential SHS as well as participating in an organized team sport (*p* < 0.05 for each characteristic). BMI at age 10 years was associated with baseline overweight/obesity status, race/ethnicity, parental education, Spanish questionnaire, residential SHS, and participating in an organized team sport (*p* < 0.05 for each characteristic). The correlation between *in utero* and childhood freeway NRAP exposures was 0.35 (*p* < 0.0001) and the correlation between the first year of life and childhood freeway NRAP exposures was 0.58 (*p* < 0.0001) (Additional file 1).

Because our analysis was limited to movers only, comparison of baseline characteristics of movers and non-movers can be found in Additional file 2. Movers were slightly older at baseline (6.5 years, SD = 0.7) compared to non-movers (6.3 years, SD = 0,7). Racial/ethnics groups differed between movers and non-movers with more Hispanic children in the non-movers group, 63% versus 56% in movers. Movers had more parents with an above high school education (62%) compared to non-movers (51%) and non-movers had more parents who filled out the baseline questionnaire in Spanish (37%) than movers (22%). Movers had a higher participation in organized team sports (42%) than non-movers (34%). Movers and non-movers did not differ in obesity status, sex, self-reported premature birth, maternal smoking during pregnancy, residential second-hand smoke, and lifetime history of asthma.

Residential NRAP exposures measured in near-road NO_x_ for freeway and non-freeway sources for *in utero*, first year of life and childhood periods are described in Table 2.

**Table 2:** Residential NRAP exposures from freeway and non-freeway sources for *in utero*, first year of life, and childhood periods in children in the CHS

Exposure Period	Mean ± SD	Median	IQR	Range
Freeway NO_x_ (ppb)				
*In utero*	16.7 ± 20.1	10.8	4.4–22.1	0–233.3
First year of life	16.2 ± 19.5	10.4	4.1–21.9	0–304.2
Childhood	15.1 ± 18.9	9.6	4.6–18.1	0–351.0
Non-Freeway NO_x_ (ppb)				
*In utero*	10.3 **±** 7.4	8.7	5.2–13.7	0.0003–74.0
First year of life	9.3 **±** 6.7	7.9	4.6–12.2	0.0003–77.4
Childhood	6.2 **±** 4.7	5.2	3.2–7.6	0.09–65.7

Mean near-road NO_x_ exposures from freeway roadways during *in utero*, first year of life, and childhood were 16.7 parts per billion (ppb) (SD: 20.1), 16.2 ppb (SD: 19.5), and 15.1 ppb (SD: 18.9), and mean near-road NO_x_ exposures from non-freeway roadways during* in utero*, first year of life, and childhood were 10.3 ppb (SD: 7.4), 9.3 ppb (SD: 6.7), and 6.2 ppb (SD: 4.7), respectively.

### Associations of early life NRAP exposures with childhood BMI

First year of life exposures to NRAP from freeway roads were positively associated with BMI at age 10 years and BMI growth during study follow up and these associations were independent of mid-childhood NRAP exposures (Table 3).

**Table 3:** Effects of *in utero*/first year of life and childhood *freeway* NO_x_ exposures on 4-year childhood BMI trajectories in CHS children

Freeway NO_x_ (ppb)	BMI Growth Per Year^a^ Effect (95% CI)	BMI at Age 10 Years^a^ Effect (95% CI)
Model 1		
*In utero* (*n* = 2072)	0.05 (−0.02, 0.1)	0.1 (− 0.3, 0.5)
Childhood	−0.02 (− 0.1, 0.05)	0.05 (− 0.4, 0.5)
Model 2		
First year of life (n = 2318)	0.1 (0.03, 0.2)*	0.5 (0.02, 0.9)*
Childhood	− 0.06 (− 0.1, 0.02)	−0.1 (− 0.6, 0.3)

^a^BMI growth (kg/m^2^) over study follow up and difference in attained BMI at age 10 years scaled to 2 standard deviations of *in utero *freeway NOx exposure with 40.1 ppb, first year of life freeway NOx with 39.1 ppb, and childhood freeway NOx with 37.8 ppb

*p < 0.05

For first year of life (model 2), a 39.1 ppb difference in near-road freeway NO_x_ exposure was significantly associated with a 0.1 kg/m^2^ (95% confidence interval (CI): 0.03, 0.2) faster increase in BMI per year resulting in a 0.5 kg/m^2^ (95% CI: 0.02, 0.9) higher BMI at age 10 years. For *in utero* (model 1), a 40.1 ppb difference in near-road freeway NO_x_ exposure was associated with a 0.05 kg/m^2^ (95% CI: -0.02, 0.1) faster increase in BMI per year and a 0.1 kg/m^2^ (95% CI: -0.3, 0.5) higher BMI at age 10 years; however, these estimates did not reach statistical significance after adjusting for confounders (Table 3).

In contrast, non-freeway NRAP exposures during mid-childhood were associated with BMI at age 10 years and BMI growth while early life non-freeway NRAP exposures showed no significant association (Table 4). Additionally, near-road total NO_x_ exposures were similar in magnitude to that of near-road freeway NOx exposures (Additional file 3).

**Table 4:** Effects of *in utero*/first year of life and childhood *non-freeway* NO_x_ exposures on 4-year childhood BMI trajectories in children in CHS

Non-Freeway NO_x_ (ppb)	BMI Growth Per Year^a^ Effect (95% CI)	BMI at Age 10 Years^a^ Effect (95% CI)
Model 1		
*In utero* (*n* = 2072)	0.03 (−0.05, 0.1)	0.1 (− 0.3, 0.6)
Childhood	0.08 (−0.007, 0.2)	0.6 (0.08, 1.03)*
Model 2		
First year of life (n = 2318)	− 0.02 (− 0.1, 0.06)	−0.07 (− 0.5, 0.4)
Childhood	0.1 (0.01, 0.2)*	0.6 (0.1, 1.1)*

^a^BMI growth (kg/m^2^) over study follow up and difference in attained BMI at age 10 years scaled to 2 standard deviations of *in utero* non-freeway NOx with 14.7 ppb, first year of life non-freeway NOx with 18.7 ppb, and childhood non-freeway NOx with 9.4 ppb

*p < 0.05

Based on tests for interaction and the analysis stratified by effect modifiers, there was little evidence to support differences in effects of early life freeway NRAP exposure by sex (males versus females), race/ethnicity (Non-Hispanic Whites versus Hispanics), and baseline overweight/obese status (overweight/obese versus normal BMI) (Additional files 4, 5 and 6). In a subsample of children who had complete data of birth weight and gestational age (*n* = 2129), birth weight and gestational age did not significantly change the effects of *in utero* or first year of life near-road freeway NO_x_ exposure on BMI growth and BMI at age 10 years (Additional file 7). We also explored effects of *in utero* and first year of life ambient PM_2.5_ exposures on childhood BMI trajectory however we did not see any significant associations (Additional file 8). We conducted further sensitivity analysis comparing independent contributions of *in utero* or first year of life near-road freeway NO_x_ exposures without adjustments of mid-childhood exposures on BMI trajectory for movers and non-movers to assess possibility of selection bias (Additional file 9). We found similar growth trajectories amongst movers and non-movers when looking at *in utero* or first year of life NRAP exposures.

## Discussion

In our study population of school-aged children in Southern California, higher first year of life NRAP from freeway sources were associated with faster increases in BMI during childhood after adjusting for confounders such as age, sex, race/ethnicity, parental education, Spanish questionnaire, and mid-childhood NRAP exposures. These longitudinal associations were independent of mid-childhood NRAP exposures and resulted in significant differences in BMI at age 10 years, suggesting that early life NRAP exposures may represent important windows of exposure that increase risk for developing childhood obesity. Previous studies of this cohort have shown that increased mid-childhood NRAP was associated with increased BMI growth where children in the highest 10% of non-freeway NO_x_ exposure showed a 0.39 kg/m^2^ higher BMI at age 10 years and a 0.087 kg/m^2^ faster increase in BMI per year when compared to those in the lowest 10% of exposure using a similar modeling approach [9]. We found consistent results as those reported in Jerrett et al. 2014 using our childhood, non-freeway NRAP exposure in our subset of children with longitudinal BMI measures, movers only, as well as movers with *in utero* NRAP exposures, and movers with first year of life NRAP exposures suggesting that the NRAP association were not the result of selecting movers for this study (Additional file 10).

Collectively, these results suggest that specific components of NRAP exposure in freeway versus non-freeway mixtures may independently contribute to obesity risk during different periods of early life and childhood. Vehicle exhaust is the main contributor to NRAP and chemical composition of freeway and non-freeway roads have shown to be different due to differences in vehicle types and vehicle volume [29]. For example, heavy duty diesel trucks with compression ignition engines travel most densely on freeways compared to non-freeway roads particularly in Southern California, and diesel truck emissions also differ from spark ignition engine emissions which are primarily gasoline derived passenger vehicles. Furthermore, a study in Texas also showed that three different road types had notable differences in chemical composition due to the varying vehicle types and emissions [30, 31]. Due to differences in total volume and emissions from diesel engine versus gasoline engine vehicles, chemical composition downwind of freeway and non-freeway roadways is expected to differ.

Our findings build on previous work in animal studies that have reported associations of early life air pollution exposures and obesity [32, 33]. In mice, *in utero* exposure to diesel exhaust predisposed offspring to higher weight gain when fed a high fat diet compared to those offspring exposed to filtered air and given a high fat diet [32]. Additionally, Sprague Dawley rats exposed to unfiltered Beijing air pollution prenatally and continuously after birth had significantly higher fat mass at 8 weeks [33]. Together, results from our study coupled with animal models suggest that early life exposures may represent a critical window of exposure where increased NRAP may result in increased risk for higher childhood BMI trajectories, which in turn may lead to childhood obesity.

The biological mechanisms linking air pollution exposure with increased childhood BMI remain uncertain. However, animal models suggest inflammatory pathways where increased air pollution exposures have been shown to result in higher levels of circulating proinflammatory cytokines, inflammation in the lungs, lower levels of anti-inflammatory cytokines as well as adipose tissue inflammation [33–35]. Exposures may also have effects on the brain via neuroinflammation where increased *in utero* air pollution exposure was shown to stimulate appetite or anxiety induced over-eating in mice [32]. Additionally, animal models suggest that increased polycyclic aromatic hydrocarbon exposure, a byproduct of traffic combustion, may increase white adipose tissue accumulation and inhibit lipolysis [36, 37]. These animal studies have uncovered some of the potential mechanisms underlying the associations between increased *in utero* and early life NRAP exposures with obesity. Our study builds on this work by showing longitudinal associations between early life NRAP exposure and BMI growth in children 6–10 years of age.

Despite the strengths of this study, dietary data was unavailable and residual confounding may have occurred since poor diet, such as increased sugar-sweetened beverage consumption, is associated with increased risk of childhood obesity [38, 39] and NRAP exposure through lower socioeconomic position [40]. However, the models adjusted for important covariates that may be related to these factors and it is unlikely that results are fully explained by residual confounding. The current study used residential based estimates of NRAP exposure with near-road NO_x_ concentrations as a marker of NRAP exposures. While exposure misclassification may have occurred, misclassification should be random amongst subjects therefore biasing estimates toward the null. This study was also limited to BMI from annual height and weight measures since direct measures of adiposity were not performed [41]. Lastly, since *in utero* and first year of life NRAP exposures were highly correlated, we are unable to conclude the relative contribution of each exposure window (i.e.,* in utero *versus first year of life) to increased childhood BMI.

## Conclusions

Our results show that increased first year of life near-road freeway NO_x_ exposures are associated with increased velocity of childhood BMI growth trajectory and higher attained BMI at 10 years and remained robust after controlling for multiple confounders as well as childhood near-road freeway NO_x_ exposures. Furthermore, increased childhood near-roadway exposures from non-freeway sources were associated with increased BMI growth and higher BMI at 10 years, consistent with our past findings. These results, along with other epidemiological and animal studies, implicate environmental exposures such as NRAP as potential risk factors for higher childhood BMI growth and higher attained BMI at age 10 years. These findings have significant public health relevance for intervention since the number of children living near freeways and busy roadways is large and continues to increase [42]. Additional epidemiological and experimental studies are warranted to examine the mechanisms by which early life NRAP exposures may impact early growth trajectories in children.

## Additional files

Additional file 1: Pearson correlation coefficients between *in utero*/first year of life and childhood NRAP exposure in non-movers and movers in children in the Children’s Health Study. (DOCX 14 kb)

Additional file 2: Baseline characteristics of movers and non-movers with early life NRAP exposures and who were enrolled in the longitudinal Children’s Health Study^a. (DOCX 18 kb)^

Additional file 3: Effects of *in utero*/first year of life and childhood near-road total NO_x_^a^ exposure on 4-year childhood BMI trajectories. (DOCX 14 kb)

Additional file 4: Effects of *in utero*/first year of life near-road freeway NO_x_ on 4-year childhood BMI trajectories for male and female children. (DOCX 15 kb)

Additional file 5: Effects of *in utero*/first year of life near-road freeway NO_x_ on 4-year childhood BMI trajectories for Non-Hispanic White/Hispanic children. (DOCX 15 kb)

Additional file 6: Effects of *in utero*/first year of life near-road freeway NO_x_ on 4-year childhood BMI trajectories for baseline overweight/obese and normal BMI children. (DOCX 15 kb)

Additional file 7: Effects of *in utero*/first year of life near-road freeway NO_x_ on 4-year childhood BMI trajectories adjusting for birth weight and gestational age. (DOCX 14 kb)

Additional file 8: Effects of *in utero*/first year of life and childhood ambient PM_2.5_ exposure on 4-year childhood BMI trajectories. (DOCX 14 kb)

Additional file 9: Independent effects of *in utero* or first year of life near-road freeway NO_x_ exposure on 4-year childhood BMI trajectories in movers and non-movers. (DOCX 15 kb)

Additional file 10: Effects of childhood, near-road non-freeway NO_x_ exposure on 4-year childhood BMI trajectories for children enrolled in the Children’s Health Study. (DOCX 14 kb)

## Abbreviations

BMI: Body mass index
CALINE4: California line-source dispersion model
CDC: Centers for Disease Control and Prevention
CHS: Children’s Health Study
CI: Confidence interval
IQR: Interquartile range
LBW: Low birth weight
NO_x_: Nitrogen oxides
NRAP: Near-roadway air pollution
PM_2.5_: Fine particulate matter ≤2.5 μm in diameter
ppb: Parts per billion
SD: Standard deviation
SHS: Secondhand smoke
