# Supplementary material for: Longitudinal associations of in utero and early life near-roadway air pollution with trajectories of childhood body mass index
Source: Environ Health. 2018 Sep 14;17:64. doi: 10.1186/s12940-018-0409-7 (PMC6137930; doi:10.1186/s12940-018-0409-7)
Supplement: Supplementary file 1 — Pearson correlation coefficients between in utero/first year of life and childhood NRAP exposure in non-movers and movers in children in the Children’s Health Study. (DOCX 14 kb) [file 12940_2018_409_MOESM1_ESM.docx]

**Additional file 1.** Pearson correlation coefficients between *in utero*/first year of life and childhood NRAP exposure in non-movers and movers in children in the Children’s Health Study.

|  | **Non-Movers^a^** | |  | **Movers^b^** | |
| --- | --- | --- | --- | --- | --- |
| **NRAP Exposure Period**  (Freeway sources) | *In Utero* | First Year of Life |  | *In Utero* | First Year of Life |
| Childhood | 0.97* | 0.98* |  | 0.35* | 0.58* |
| **NRAP Exposure Period**  (Non-freeway sources) | *In Utero* | First Year of Life |  | *In Utero* | First Year of Life |
| Childhood | 0.98* | 0.98* |  | 0.57* | 0.65* |

^a^ Non-movers were children who did not have a change in address or moved less than 500 meters between *in utero* period and study entry.

^b^ Movers were children who had a change in address between *in utero* period and CHS study entry that resulted in a move greater than or equal to 500 meters.

*p<.0001
