# Supplementary material for: Longitudinal associations of in utero and early life near-roadway air pollution with trajectories of childhood body mass index
Source: Environ Health. 2018 Sep 14;17:64. doi: 10.1186/s12940-018-0409-7 (PMC6137930; doi:10.1186/s12940-018-0409-7)
Supplement: Supplementary file 2 — Baseline characteristics of movers and non-movers with early life NRAP exposures and who were enrolled in the longitudinal Children’s Health Studya. (DOCX 18 kb) [file 12940_2018_409_MOESM2_ESM.docx]

**Additional file 2.** Baseline characteristics of movers and non-movers with early life NRAP exposures and who were enrolled in the longitudinal Children’s Health Study^a^.

| **Characteristic** | **Movers^b^** |  | **Non-Movers^c^** | **P-value** |
| --- | --- | --- | --- | --- |
|  | **No. (%)^a^** |  | **No. (%)^a^** |  |
| Obesity Status |  |  |  | 0.1 |
| Normal weight | 1651 (71.2) |  | 605 (67.5) |  |
| Overweight | 320 (13.8) |  | 142 (15.9) |  |
| Obese | 347 (15.0) |  | 149 (16.6) |  |
| Sex |  |  |  | 0.3 |
| Female | 1145 (49.4) |  | 462 (51.6) |  |
| Male | 1173 (50.6) |  | 434 (48.4) |  |
| Race/Ethnicity |  |  | | <0.0001 |
| White | 771 (33.3) |  | 223 (24.9) |  |
| Hispanic | 1290 (55.7) |  | 564 (63.0) |  |
| Black | 72 (3.1) |  | 27 (3.0) |  |
| Asian/Pacific Islander | 67 (2.9) |  | 34 (3.8) |  |
| Other | 114 (4.9) |  | 41 (4.6) |  |
| Parental Education |  |  |  | <0.0001 |
| Less than high school | 410 (18.4) |  | 249 (30.4) |  |
| High school | 430 (19.3) |  | 149 (18.2) |  |
| Above high school | 1389 (62.3) |  | 420 (51.3) |  |
| Spanish Questionnaire^d^ |  |  |  |  |
| No | 1818 (78.4) |  | 562 (62.7) | <0.0001 |
| Yes | 500 (21.6) |  | 334 (37.3) |  |
| Self-reported premature birth |  |  |  | 0.1 |
| No | 2003 (88.9) |  | 763 (90.9) |  |
| Yes | 251 (11.1) |  | 76 (9.1) |  |
| Maternal smoking during pregnancy |  |  |  | 0.8 |
| No | 2083 (92.7) |  | 770 (92.7) |  |
| Yes | 164 (7.3) |  | 61 (7.3) |  |
| Residential second hand smoke^e^ |  |  |  | 0.9 |
| No | 2110 (93.2) |  | 784 (92.8) |  |
| Yes, when child is home | 110 (4.9) |  | 41 (4.9) |  |
| Yes, when child is not home | 44 (1.9) |  | 20 (2.3) |  |
| Life-time history of asthma |  |  |  | 0.1 |
| No | 1928 (85.1) |  | 744 (87.1) |  |
| Yes | 338 (14.9) |  | 110 (12.9) |  |
| Organized team sport^f^ |  |  |  | <0.0001 |
| No | 1141 (57.8) |  | 449 (66.3) |  |
| Yes | 832 (42.2) |  | 228 (33.7) |  |

^a^ This analysis includes a subset of the Children’s Health Study participants who had available NRAP exposure data for *in utero* or first year of life periods. Movers were defined as those who had a change in address between *in utero* period and study entry that resulted in a move ≥ 500 meters. Non-movers were those who did not move > 500 meters before study entry.

^b^ First observation of participants with NRAP exposures in movers (n=2318); variable denominators may differ due to missing values.

^c^ First observation of participants with NRAP exposures in non-movers (n=896); variable denominators may differ due to missing values.

^d^ Spanish Questionnaire is if parent filled out baseline questionnaire in Spanish and serves as a surrogate measure for recent immigration.

^e^ Residential second-hand smoke is if anyone living in the child’s home smokes daily inside the home.

^f^ Organized team sport is if the child played outdoors in any organized team sport at least twice a week during the past year.
