# Supplementary material for: Longitudinal associations of in utero and early life near-roadway air pollution with trajectories of childhood body mass index
Source: Environ Health. 2018 Sep 14;17:64. doi: 10.1186/s12940-018-0409-7 (PMC6137930; doi:10.1186/s12940-018-0409-7)
Supplement: Supplementary file 3 — Effects of in utero/first year of life and childhood near-road total NOxa exposure on 4-year childhood BMI trajectories. (DOCX 14 kb) [file 12940_2018_409_MOESM3_ESM.docx]

**Additional file 3.** Effects of *in utero*/first year of life and childhood near-road total NO_x_^a^ exposure on 4-year childhood BMI trajectories.

| **Total NO_x_ Exposure (ppb)** | **BMI Growth Per Year^b^**  Effect (95% CI) | | **BMI at Age 10 Years^b^**  Effect (95% CI) |
| --- | --- | --- | --- |
| *In utero* (n=2,072) | | 0.06 (-0.009, 0.1) | 0.2 (-0.2, 0.6) |
| Childhood | | -0.009 (-0.09, 0.07) | 0.2 (-0.3, 0.6) |
| First year of life (n=2,318) | | 0.1 (0.03, 0.2)* | 0.5 (0.02, 0.9)* |
| Childhood | | -0.04 (-0.1, 0.04) | -0.05 (-0.5, 0.4) |

^a^ Total NO_x_= Freeway + Non-freeway NO_x_

^b^BMI growth and BMI at age 10 years scaled to 2 standard deviations of *in utero* total NOx exposure with 46.1 ppb, first year of life total NOx with 44.9 ppb and childhood total NOx with 42.2 ppb. Models adjusted for age, sex, race/ethnicity, parental education, and Spanish questionnaire.

*p<0.05.
