# Supplementary material for: Longitudinal associations of in utero and early life near-roadway air pollution with trajectories of childhood body mass index
Source: Environ Health. 2018 Sep 14;17:64. doi: 10.1186/s12940-018-0409-7 (PMC6137930; doi:10.1186/s12940-018-0409-7)
Supplement: Supplementary file 4 — Effects of in utero/first year of life near-road freeway NOx on 4-year childhood BMI trajectories for male and female children. (DOCX 15 kb) [file 12940_2018_409_MOESM4_ESM.docx]

**Additional file 4.** Effects of *in utero*/first year of life near-road freeway NO_x_ on 4-year childhood BMI trajectories for male and female children.

| **Freeway NO_x_ Exposure (ppb)** | **BMI growth per year^a^**  Effect (95% CI) | |  | **BMI at age 10 years^a^**  Effect (95% CI) | |
| --- | --- | --- | --- | --- | --- |
|  | Male | Female |  | Male | Female |
| *In utero*^b^ | 0.08 (-0.01, 0.2) | 0.009 (-0.09, 0.1) |  | 0.4 (-0.2, 0.9) | -0.07 (-0.6, 0.5) |
| First year of life^c^ | 0.1 (0.02, 0.2)* | 0.07 (-0.02,0.2) |  | 0.7 (0.07, 1.1)* | 0.2 (-0.5, 0.8) |

^a^ BMI growth and BMI at age 10 years scaled to 2 standard deviations of *in utero* near-road freeway NO_x_ exposure with 40.1ppb and first year of life near-road freeway NO_x_ with 39.1 ppb. Models adjusted for age, race/ethnicity, parental education, Spanish questionnaire, and childhood near-road freeway NOx exposure.

^b^ *In utero* model, males=1057, females=1014.

^c^ First year of life model, males=1173, females=1145.

*p<0.05

Interaction p-values for in utero: p_interaction BMI growth_=0.05, p_interaction BMI at age 10_=0.19

Interaction p-values for first year of life: p_interaction BMI growth_=0.062, p_interaction BMI at age 10_=0.25
