# Supplementary material for: Longitudinal associations of in utero and early life near-roadway air pollution with trajectories of childhood body mass index
Source: Environ Health. 2018 Sep 14;17:64. doi: 10.1186/s12940-018-0409-7 (PMC6137930; doi:10.1186/s12940-018-0409-7)
Supplement: Supplementary file 5 — Effects of in utero/first year of life near-road freeway NOx on 4-year childhood BMI trajectories for Non-Hispanic White/Hispanic children. (DOCX 15 kb) [file 12940_2018_409_MOESM5_ESM.docx]

**Additional file 5.** Effects of *in utero*/first year of life near-road freeway NO_x_ on 4-year childhood BMI trajectories for Non-Hispanic White/Hispanic children.

| **Freeway NO_x_ Exposure (ppb)** | **BMI growth per year^a^**  Effect (95% CI) | |  | **BMI at age 10 years^a^**  Effect (95% CI) | |
| --- | --- | --- | --- | --- | --- |
|  | Non-Hispanic White | Hispanic |  | Non-Hispanic White | Hispanic |
| *In utero*^b^ | 0.09 (-0.03, 0.2) | 0.01 (-0.07, 0.1) |  | 0.5 (-0.2, 1.1) | -0.07 (-0.6, 0.5) |
| First year of life^c^ | 0.1 (-0.04, 0.3) | 0.08 (-0.009,0.2) |  | 0.7 (-0.1, 1.4) | 0.3 (-0.3, 0.9) |

^a^ BMI growth and BMI at age 10 years scaled to 2 standard deviations of *in utero* near-road freeway NO_x_ exposure with 40.1 ppb and first year of life with 39.1 ppb. Models adjusted for age, sex, parental education, Spanish questionnaire, and childhood near-road freeway NOx.

^b^ *In utero* model: White, n=695; Hispanic, n=1151.

^c^ First year of life: White, n=771; Hispanic n=1289.

Interaction p-values for in utero: p_interaction BMI growth_=0.078, p_interaction BMI at age 10_=0.35

Interaction p-values for first year of life: p_interaction BMI growth_=0.0.013, p_interaction BMI at age 10_=0.2
