# Supplementary material for: Longitudinal associations of in utero and early life near-roadway air pollution with trajectories of childhood body mass index
Source: Environ Health. 2018 Sep 14;17:64. doi: 10.1186/s12940-018-0409-7 (PMC6137930; doi:10.1186/s12940-018-0409-7)
Supplement: Supplementary file 6 — Effects of in utero/first year of life near-road freeway NOx on 4-year childhood BMI trajectories for baseline overweight/obese and normal BMI children. (DOCX 15 kb) [file 12940_2018_409_MOESM6_ESM.docx]

**Additional file 6.** Effects of *in utero*/first year of life near-road freeway NO_x_ on 4-year childhood BMI trajectories for baseline overweight/obese and normal BMI children.

| **Freeway NO_x_ Exposure (ppb)** | **BMI growth per year^a^**  Effect (95% CI) | |  | **BMI at age 10 years^a^**  Effect (95% CI) | |
| --- | --- | --- | --- | --- | --- |
|  | Baseline Overweight or Obese^b^ | Baseline Normal BMI^c^ |  | Baseline Overweight or Obese^b^ | Baseline Normal BMI^c^ |
| *In utero*^d^ | 0.06 (-0.07, 0.2) | 0.03 (-0.04, 0.1) |  | 0.2 (-0.5, 0.9) | 0.07 (-0.2, 0.3) |
| First year of life^e^ | 0.07 (-0.06, 0.2) | 0.08 (-0.001,0.2) |  | 0.09 (-0.6, 0.8) | 0.2 (-0.1, 0.5) |

^a^ BMI growth and BMI at age 10 years scaled to 2 standard deviations of *in utero* near-road freeway NO_x_ exposure with 40.1 ppb and first year of life NO_x_ with 39.1 ppb. Models adjusted for age, sex, race/ethnicity, parental education, Spanish questionnaire.

^b^Baseline overweight/obese= age-, sex-specific CDC BMI percentile ≥ 85.

^c^Normal BMI= age-, sex-specific CDC BMI percentile <85.

^d^ *In utero*: overweight/obese, n=591; normal, n=1480.

^e^ First year of life: overweight/obese, n=667; normal, n=1651.

Interaction p-values for in utero: p_interaction BMI growth_<0.0001, p_interaction BMI at age 10_<0.0001.

Interaction p-values for first year of life: p_interaction BMI growth_<0.0001, p_interaction BMI at age 10_<0.0001.
