# Supplementary material for: Longitudinal associations of in utero and early life near-roadway air pollution with trajectories of childhood body mass index
Source: Environ Health. 2018 Sep 14;17:64. doi: 10.1186/s12940-018-0409-7 (PMC6137930; doi:10.1186/s12940-018-0409-7)
Supplement: Supplementary file 7 — Effects of in utero/first year of life near-road freeway NOx on 4-year childhood BMI trajectories adjusting for birth weight and gestational age. (DOCX 14 kb) [file 12940_2018_409_MOESM7_ESM.docx]

**Additional file 7.** Effects of *in utero*/first year of life near-road freeway NO_x_ on 4-year childhood BMI trajectories adjusting for birth weight and gestational age.

| **Freeway NO_x_**  **Exposure (ppb)** | **BMI Growth Per Year^a^**  Effect (95% CI) | **BMI at Age 10 Years^a^**  Effect (95% CI) |
| --- | --- | --- |
| *In utero* (n=1,926) | 0.06 (-0.01, 0.1) | 0.2 (-0.2, 0.6) |
| First year of life (n=2,129) | 0.1 (0.03, 0.2)* | 0.5 (0.04, 0.9)* |

^a^ BMI growth and BMI at age 10 years scaled to 2 standard deviations of *in utero* near-road freeway NO_x_ exposure with 40.1 ppb and first year of life NO_x_ with 39.1 ppb. Models adjusted for age, sex, race/ethnicity, parental education, Spanish questionnaire, childhood near-road freeway NO_x_ exposure, birth weight (grams), and gestational age (days).

*p<0.05.
