# Supplementary material for: Longitudinal associations of in utero and early life near-roadway air pollution with trajectories of childhood body mass index
Source: Environ Health. 2018 Sep 14;17:64. doi: 10.1186/s12940-018-0409-7 (PMC6137930; doi:10.1186/s12940-018-0409-7)
Supplement: Supplementary file 8 — Effects of in utero/first year of life and childhood ambient PM2.5 exposure on 4-year childhood BMI trajectories. (DOCX 14 kb) [file 12940_2018_409_MOESM8_ESM.docx]

**Additional file 8.** Effects of *in utero*/first year of life and childhood ambient PM_2.5_ exposure on 4-year childhood BMI trajectories.

| **Ambient PM_2.5_**  **Exposure (ug/m^3^)** | **BMI Growth Per Year^a^**  Effect (95% CI) | **BMI at Age 10 Years^a^**  Effect (95% CI) |
| --- | --- | --- |
| *In utero* (n=2,524) | -0.06 (-0.1, 0.02) | -0.6 (-1.1, -0.1) |
| Childhood | -0.03 (-0.1, 0.06) | 0.3 (-0.1, 0.8) |
| First year of life (n=2,621) | 0.03 (-0.1, 0.05) | -0.5 (-0.9, -0.02) |
| Childhood | -0.05 (-0.1, 0.04) | 0.2 (-0.2, 0.7) |

^a^ BMI growth and BMI at age 10 years scaled to 2 standard deviations of *in utero* ambient PM_2.5_ exposure with 17.0 ug/m^3^, first year of life total NOx with 14.8 ug/m^3^ and childhood with 9.9 ug/m^3^. Models adjusted for age, sex, race/ethnicity, parental education, and Spanish questionnaire.
