# Supplementary material for: Longitudinal associations of in utero and early life near-roadway air pollution with trajectories of childhood body mass index
Source: Environ Health. 2018 Sep 14;17:64. doi: 10.1186/s12940-018-0409-7 (PMC6137930; doi:10.1186/s12940-018-0409-7)
Supplement: Supplementary file 9 — Independent effects of in utero or first year of life near-road freeway NOx exposure on 4-year childhood BMI trajectories in movers and non-movers. (DOCX 15 kb) [file 12940_2018_409_MOESM9_ESM.docx]

**Additional file 9.** Independent effects of *in utero* or first year of life near-road freeway NO_x_ exposure on 4-year childhood BMI trajectories in movers and non-movers.

| **Freeway NO_x_ Exposure (ppb)** | **BMI growth per year^a^**  Effect (95% CI) | |  | **BMI at age 10 years^a^**  Effect (95% CI) | |
| --- | --- | --- | --- | --- | --- |
|  | Movers | Non-movers |  | Movers | Non-movers |
| *In utero* only^b^ | 0.04  (-0.03, 0.1) | 0.05  (-0.05, 0.1) |  | 0.2  (-0.2, 0.5) | 0.7  (0.07, 1.3)* |
| First year of life only^c^ | 0.07  (0.01, 0.1)* | 0.04  (-0.06, 0.1) |  | 0.4  (0.03, 0.7)* | 0.6  (0.02, 1.2)* |

^a^ BMI growth and BMI at age 10 years scaled to 2 standard deviations: for movers *in utero* freeway NOx exposure with 40.1 ppb, first year of life freeway NOx with 39.1 ppb; for non-movers *in utero* freeway NOx exposure with 42.6 ppb, first year of life freeway NOx with 41.3 ppb. Models adjusted for age, sex, race/ethnicity, parental education, and Spanish questionnaire.

^b^ *In utero* model, movers=2,072; non-movers=884.

^c^ First year of life movers=2,318; non-movers=896.

*p<0.05.
