# Supplementary material for: Longitudinal associations of in utero and early life near-roadway air pollution with trajectories of childhood body mass index
Source: Environ Health. 2018 Sep 14;17:64. doi: 10.1186/s12940-018-0409-7 (PMC6137930; doi:10.1186/s12940-018-0409-7)
Supplement: Supplementary file 10 — Effects of childhood, near-road non-freeway NOx exposure on 4-year childhood BMI trajectories for children enrolled in the Children’s Health Study. (DOCX 14 kb) [file 12940_2018_409_MOESM10_ESM.docx]

**Additional file 10.** Effects of childhood, near-road non-freeway NO_x_ exposure on 4-year childhood BMI trajectories for children enrolled in the Children’s Health Study.

| **Childhood Non-freeway NO_x_ Exposure (ppb)** | **BMI growth** Effect (95% CI) | **BMI at age 10 years** Effect (95% CI) |
| --- | --- | --- |
| Model 1: Subjects with 2+ BMI Measures | 0.06 (0.02, 0.1)* | 0.4 (0.2, 0.7)* |
| Model 2: Movers only | 0.07 (0.02, 0.1)* | 0.6 (0.3, 0.9)* |
| Model 3: Subjects with in utero exposures | 0.07 (0.02, 0.1)* | 0.5 (0.1, 0.8)* |
| Model 4: Subjects with first year of life exposures | 0.07 (0.02, 0.1)* | 0.4 (0.1, 0.8)* |

^a^ BMI growth and BMI at age 10 years scaled to 2 standard deviations of childhood near-road non-freeway NOx exposure with 9.4 ppb. Models adjusted for age, sex, race/ethnicity, parental education, and Spanish questionnaire.

*p<0.05.
